# Case Report: Low-dose radiotherapy as part of multimodal salvage therapy for immunotherapy-resistant SMARCA4-deficient non‐small cell lung cancer

**DOI:** 10.3389/fonc.2026.1941008

**Published:** 2026-10-05

**Authors:** Tong Bu, Nan Zhang, Tong Jin, Zixuan Wang, Yichen Mao, Dan Zong, Wenjie Guo, Xia He

**Affiliations:** 1 Department of Radiation Oncology, The Affiliated Cancer Hospital of Nanjing Medical University, Jiangsu Cancer Hospital and Jiangsu Institute of Cancer Research, Nanjing, China; 2 Jiangsu Key Laboratory of Innovative Cancer Diagnosis & Therapeutics, Nanjing, China

**Keywords:** immune modulation, immunotherapy, low-dose radiotherapy, primary resistance, SD-NSCLC

## Abstract

SMARCA4-deficient non-small cell lung cancer (SD-NSCLC) is a highly aggressive molecular subtype. This malignancy is often diagnosed at an advanced stage, carrying a dismal prognosis and also demonstrating poor responsiveness to traditional treatment. Beyond its primary site, the disease can exhibit unusual metastatic patterns such as metastasis to oral soft tissues. We report a unique case of a 61-year-old male heavy smoker with molecularly confirmed SD-NSCLC who experienced rapid metastatic progression to the right gingiva and neck lymph nodes shortly after radical surgery and initial chemo-immunotherapy. This clinical scenario exemplifies primary resistance to immune checkpoint inhibitors (ICIs), where pre-existing genetic or microenvironmental barriers hinder initial therapeutic success. To overcome this resistance, a salvage multimodal strategy was implemented, integrating ICIs with etoposide/cisplatin (EP) chemotherapy and low-dose radiotherapy (LDRT). Remarkably, this regimen achieved significant regression of the bulky metastatic lesions and has maintained sustained disease stability for over 15 months, with the patient remaining clinically stable as of the latest follow-up in June 2026. This case may hint the potential role of LDRT as an immunomodulatory component to overcome primary resistance in SD-NSCLC, suggesting a potential therapeutic strategy for patients who experience disease progression following first-line chemo-immunotherapy.

## Introduction

Lung cancer continues to be a leading cause of global cancer-related mortality, with epigenetic dysregulation increasingly recognized as a key driver of its aggressive behavior. The Switch/Sucrose Non-Fermentable (SWI/SNF) complex is an evolutionarily conserved, ATPase-dependent chromatin remodeling unit that regulates gene transcription, cellular differentiation, and DNA repair. SMARCA4 (also known as BRG1) serves as a catalytic subunit of this complex, utilizing ATP hydrolysis to manipulate nucleosome positioning. NSCLC with SMARCA4 deficiency is an uncommon tumor subtype, representing about 10% of all NSCLC cases (1). SD-NSCLC typically presents in older male smokers and is characterized by a poorly differentiated or solid adenocarcinoma histology that lacks common targetable mutations such as EGFR or ALK (2). SD-NSCLC exhibits highly aggressive behavior and a dismal prognosis in clinical, with a reported median overall survival of approximately 12.2 months, significantly shorter than other NSCLC (2).

This manuscript presents a unique case of SD-NSCLC with rare gingival metastasis, in which disease control was achieved after multimodal treatment incorporating LDRT following early progression after initial immunotherapy. To our knowledge, no published preclinical or clinical study has specifically evaluated LDRT in SD-NSCLC. By contextualizing this clinical outcome with a targeted review of current evidence, we provide a focused discussion of the diagnosis of SD-NSCLC, rare metastatic patterns, and the potential role of LDRT as an immunomodulatory component in multimodal salvage therapy for this challenging molecular subtype.

## Methods

This study was designed as a retrospective single-case analysis combined with a targeted literature review. The clinical, imaging, pathological, treatment and follow-up data of the patient were retrieved from the electronic medical record system. To summarize the clinical features, diagnostic clues and treatment strategies of this rare presentation, we searched published case reports, case series, original articles and reviews of same primary tumors and similar metastatic sites, clinical manifestations. Conference abstracts, duplicate reports, articles with incomplete or unconfirmed data were excluded.

## Case reports

### Initial diagnosis

On November 8, 2024, a 61-year-old man with a 30-year smoking history was found to have a mass in the right upper lobe of the lung during a routine physical examination. The patient denied having a family history of tumor-related conditions. PET-CT was conducted on November 15, 2024 revealed a hypermetabolic tumor in the apical segment of the right upper lobe, measuring approximately 58*45 mm with an SUV_max_ of 35.10, together with mediastinal and hilar lymph node metastasis. According to the patient’s medical records from the outside hospital, the patient underwent video-assisted thoracoscopic right upper lobectomy under general anesthesia on November 20, 2024. The lobectomy specimen measured 19 x 7 x 4 cm, and the primary tumor measured 5.8 x 5.5 x 5.0 cm. The bronchial margin showed focal tumor involvement after deeper paraffin sections. Postoperative pathology was consistent with SD-NSCLC, with metastatic involvement in 5 of 14 dissected lymph nodes. According to the 8th edition of the AJCC/UICC TNM staging system, the pathological stage was pT3N2M0 (stage IIIB). Immunohistochemistry testing showed BRG-1 negativity. CK, CK7, P53 and C-MET were positive. P63, P40, TTF-1, Syn, and Napsin A were all negative. The Ki-67 index was approximately 50%. The results of the genetic testing were also taken into account. TP53 mutations (c.C742T and c.G818T) were identified, microsatellite status was stable (MSS, MSI SCORE 8.37%), and PD-L1 TPS was 50%. The primary tumor is currently under good control (**Figures 1A–C**). The high Ki-67 index in this case may partly explain the aggressive clinical course, we will conduct a more detailed analysis in the following text.

**Figure 1:**
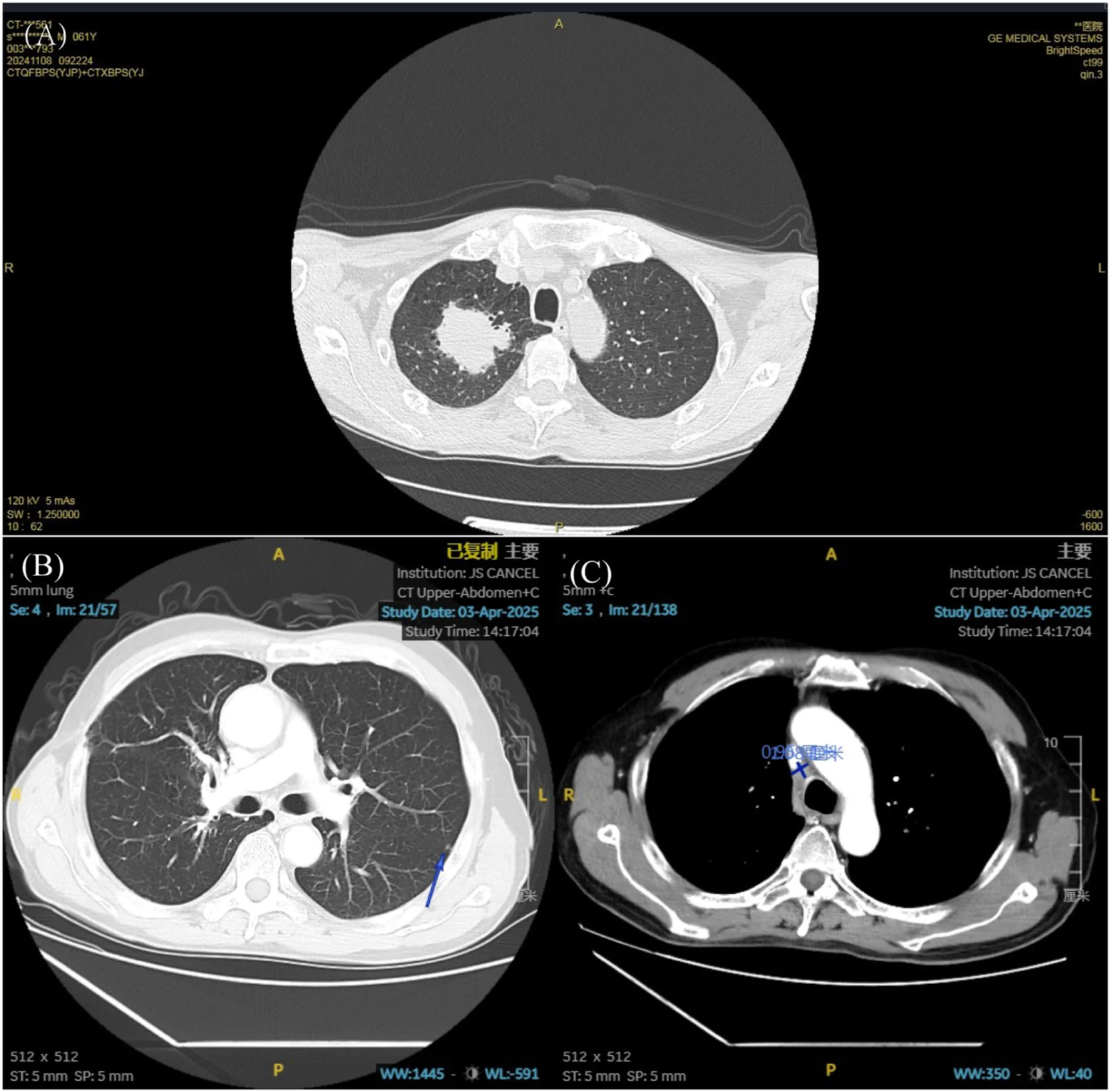
**(A)** Preoperative CT image of the primary lung lesion obtained on November 18, 2024. **(B)** Postoperative CT image of the surgical bed in the lung window obtained on April 3, 2025. **(C)** Postoperative CT image of the surgical bed in the mediastinal window obtained on April 3, 2025.

### Disease progression and multimodal treatment

On December 11, 2024, the patient started postoperative first-line chemoimmunotherapy with sintilimab, paclitaxel and carboplatin. Follow-up cervical MRI on December 25, 2024 showed an abnormal signal adjacent to the right submandibular gland. However, cervical ultrasonography on January 7, 2025 suggested that the right submandibular lesion was more likely inflammatory; therefore, no immediate intervention was undertaken, and close surveillance was continued.

After the completion of the second cycle of the original regimen on January 9, 2025, the patient suffered from an oral infection, marked the enlargement of the right submandibular and right mandibular lymph nodes, and the swelling of the right lower gingiva. Laboratory testing in other hospital suggested a mixed aerobic and anaerobic infection, so a moxifloxacin anti-infective regimen was used. When the patient came to our hospital, his oral infection had subsided a lot. But high-sensitivity C-reactive protein (CRP) showed CRP >10 mg/L; therefore, we continued the same anti-infective regimen. On January 23, 2025, the spiral CT showed soft tissue thickening of the right lower gingiva accompanied with adjacent alveolar bone resorption, suggesting an invasive lesion. And the right submandibular soft tissue mass was highly suspicious for metastases. Then a biopsy of the right lower gingival lesion was subsequently performed. According to the pathological report (pathology No. 202500345), immunohistochemistry showed CK7 positivity and BRG1 loss, with CK5/6, P63, P40, P16, S-100, and AB-PAS negativity; Ki-67 was >90%. Based on the clinical history and immunophenotype, the gingival lesion was diagnosed as metastatic SMARCA4-deficient NSCLC (**Supplementary Figure S1**). Together with the clinical history and immunohistochemical findings, the patient was confirmed metastatic SD-NSCLC.

Given the progressive of tumor was rapid and the patient’s PD-L1 TPS result, the therapeutic regimen was switched to serplulimab (300 mg), etoposide (0.17 g, d1–3), and cisplatin (40 mg, d1–3) on February 7, 2025. A previous randomized study published in JCO in 1990 showed a higher objective response rate with cisplatin/etoposide than with carboplatin/etoposide, although no overall survival difference was observed (3). Moreover, this treatment plan had already been formulated and carried out for one cycle at our superior hospital, and its efficacy had not yet been fully evaluated when the patient came to our hospital. Therefore, after clinical assessment, we continued the original regimen. However, a cervical MRI on February 25, 2025 revealed that the mass of right submandibular enlarged (5.4 × 3.5 cm) compared with the previous assessment on December 25, 2024 (**Figure 2A**). Despite the progression, radical high-dose radiotherapy was deemed unsuitable for this patient. This decision was primarily based on two factors: firstly, the submandibular mass was excessively large, and radical doses would likely cause severe collateral damage to surrounding healthy tissues; secondly, the patient had a potential concurrent oral infection, which increased the risk of radiation-induced complications. Therefore, LDRT regimen (5 Gy in 10 fractions) was initiated as an immunomodulatory rather than a purely tumor-ablative strategy.

**Figure 2:**
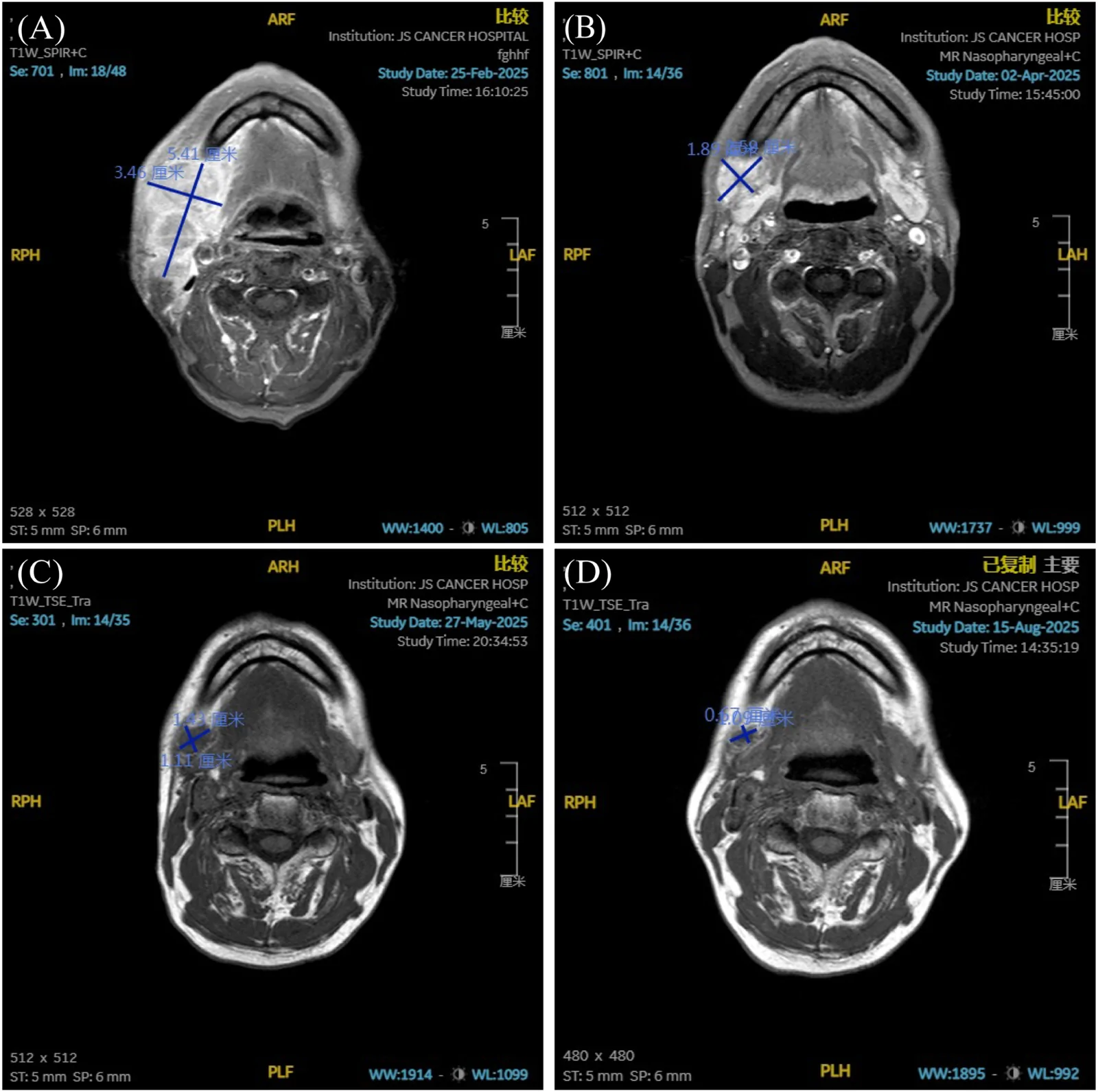
**(A)** MRI of the metastatic lesion obtained on February 25, 2025. **(B)** MRI of the metastatic lesion obtained on April 2, 2025. **(C)** MRI of the metastatic lesion obtained on May 27, 2025. **(D)** MRI of the metastatic lesion obtained on August 15, 2025.

Our patient received LDRT targeted on the neck and mediastinal lesions from March 4, 2025, with both CTV_dn_ and CTV_up_ treated with 500 cGy in 10 fractions (5 Gy/10F). On March 11, 2025, the patient completed the second cycle of the serplulimab-EC regimen. After completing two cycles of chemotherapy immunotherapy and local LDRT, the follow-up MRI conducted on April 2, 2025 showed that the mass in the right submandibular region had significantly shrunk compared to the MRI examination in February (**Figure 2B**).

Subsequently, while continuing serplulimab plus EC, the patient underwent three additional LDRT sessions targeting the right cervical and right submandibular metastatic lymph nodes on April 14, May 27 and July 9, all using the same 500 cGy in 10 fractions schedule. A follow-up cervical MRI on May 27, 2025, compared with the previous scan on April 2, 2025, revealed multiple nodular lesions exhibiting heterogeneous enhancement in the right submandibular gland region, the peri-mandibular (gingival) area, the paraparotid region, and adjacent to the right upper cervical carotid sheath. These nodules demonstrated a significant reduction in size, with the largest cross-section decreasing from approximately 2.6 × 1.9 cm to 1.4 × 1.1 cm (**Figure 2C**). Using the pre-LDRT MRI on February 25, 2025 as the baseline, the longest diameter of the mass has decreased over 70% (4). Then the MRI on August 15, 2025 indicated further shrinkage of the heterogeneously enhancing lesions at the aforementioned sites, with the maximum cross-sectional dimension decreasing from 1.4 × 1.1 cm to 1.1 × 0.6 cm (**Figure 2D**). These changes met the threshold for a PR-level decrease of this target lesion. However, because a residual measurable lesion remained on the August 15, 2025 MRI, the overall response was classified as partial response (PR) according to RECIST 1.1. After this last MRI examination, CT was chosen rather than MRI for subsequent follow-up because of financial reasons. November 28, 2025 CT showed a residual lesion with a longest diameter of 0.71 cm. Follow-up CT scans performed on March 3, 2026 and June 2, 2026 showed no measurable residual lesion, supporting an assessment of complete response (CR) and sustained disease control. Based on the assessment, this therapeutic regimen was highly effective during this clinical phase (**Supplementary Table S1**; **Supplementary Figure S2**).

Because of the favorable therapeutic response and the high intensity of the initial combination regimen, the patient was transitioned to the maintenance therapy with serplulimab monotherapy and continued to receive periodic LDRT. A total of eight courses of LDRT were administered to the localized lesions throughout the clinical course. At the follow-up in June 2026, the patient’s clinical condition remained stable, with the final administration of serplulimab occurring on January 23, 2026 (**Figure 3**).

**Figure 3:**
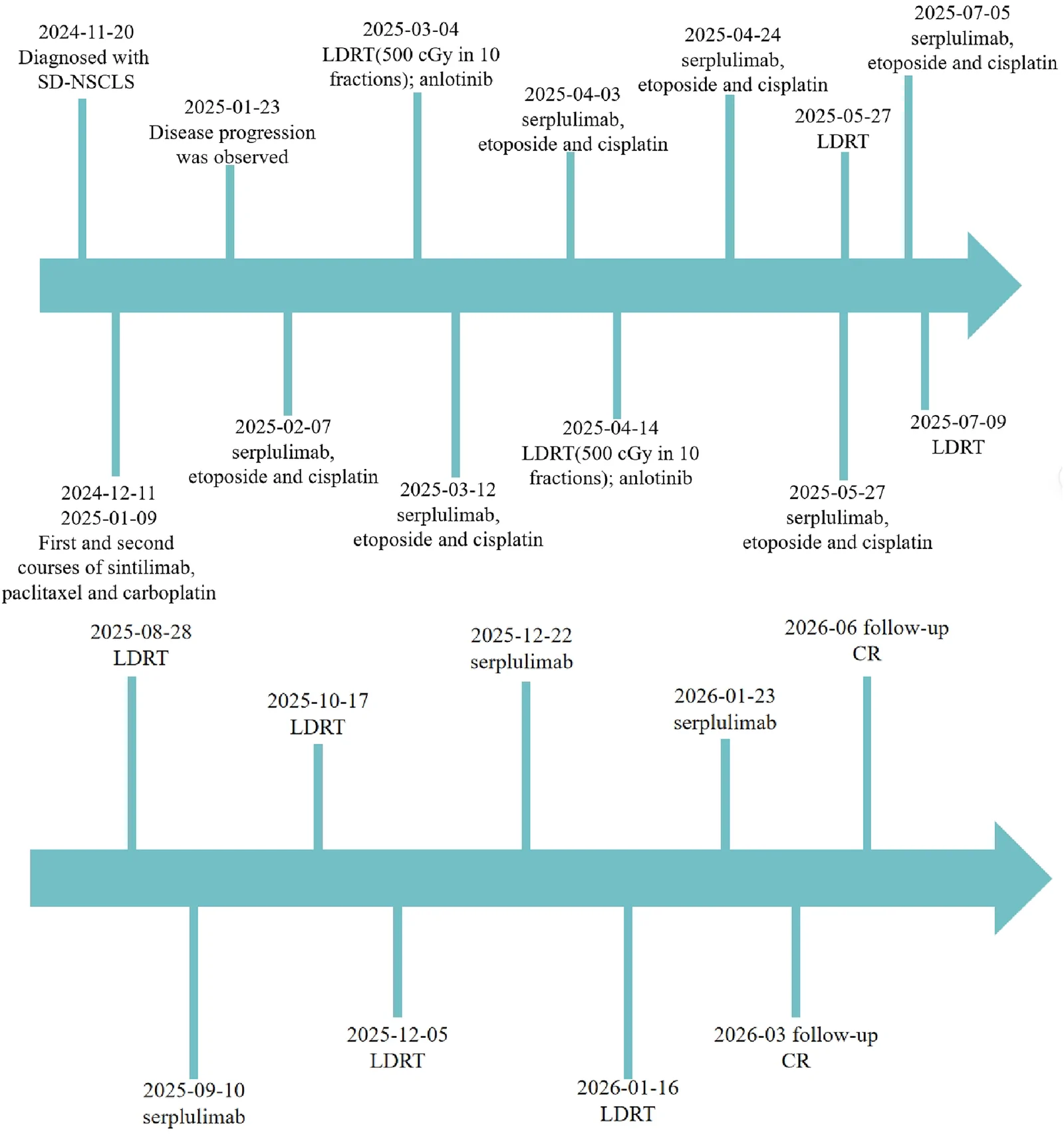
Chronological overview of the treatment course. CR, complete response.

Given that the mass of the gingival metastasis was quite large when the patient came to our hospital, so for the first LDRT course, the target volume was defined as CTV-up, covering the lymphatic drainage areas of right neck nodes levels I_a_, I_b_, and II. For the subsequent seven LDRT courses, the target volume was defined as GTV, including the metastatic lymph nodes of right neck in levels I_b_ and II. The following table presents detailed LDRT protocol (**Table 1**).

**Table 1:** Detailed information of the eight courses of low-dose radiotherapy.

LDRT course	Start date	End date	Treatment target	Radiotherapy technique	Dose per fraction (Gy)	Number of fractions (F)	Total dose (Gy)	Repeated irradiation	Interval from previous LDRT (day)
Course 1	04/03/2025	17/03/2025	cervical and mediastinal lesions	IGRT	0.5	10	5	Ia, Ib,II	NA
Course 2	14/04/2025	27/04/2025	cervical and mediastinal lesions	IGRT	0.5	10	5	Ib,II	41
Course 3	27/05/2025	10/06/2025	right cervical lymph nodes	IGRT	0.5	10	5	Ib,II	43
Course 4	09/07/2025	23/07/2025	right cervical lymph nodes	IGRT	0.5	10	5	Ib,II	43
Course 5	28/08/2025	10/09/2025	right cervical lymph nodes	IGRT	0.5	10	5	Ib,II	50
Course 6	17/10/2025	30/10/2025	right cervical lymph nodes	IGRT	0.5	10	5	Ib,II	50
Course 7	05/12/2025	18/12/2025	right submandibular lymph nodes	IGRT	0.5	10	5	Ib,II	49
Course 8	16/01/2026	30/01/2026	right cervical lymph nodes	IGRT	0.5	10	5	Ib,II	42

IGRT, image-guided radiotherapy.

## Discussion

This report adds additional evidence to the limited literature on SD-NSCLC with LDRT and rare gingival metastasis. Currently, managing metastatic NSCLC after first-line chemoimmunotherapy failure is still challenging because subsequent treatment options are limited. Standard second-line therapies, such as docetaxel (with or without anti-angiogenic agents), provide only modest survival benefits. Furthermore, several large-scale Phase III clinical trials aimed at overcoming resistance after first-line failure have recently reported negative results (5–7). Even new agents, such as the ADCs like datopotamab deruxtecan (in TROPION-Lung01) and sacituzumab govitecan (in EVOKE-01), failed to meet survival endpoints in unselected post-immunotherapy populations (8, 9).

In our patient, he experienced rapid progression after initial immunotherapy. This clinical course is consistent with “primary resistance”. According to the 2023 consensus from the Society for Immunotherapy of Cancer (SITC), primary resistance refers to a scenario where the best response to initial immune checkpoint blockade is either disease progression or stable disease lasting less than 6 months (10). So the term “primary resistance” refers specifically to the early progression observed during postoperative adjuvant chemoimmunotherapy in our case. Multiple mechanisms likely contribute to such resistance. Ongoing studies indicate that both tumor cell-intrinsic an cell-extrinsic factors contribute to primary resistance (11).

Despite potentially favorable biomarker such as high PD-L1 expression (high TPS) and TP53 mutation in this case, the tumor still rapidly progress and primary resistance may have occurred. High PD-L1 expression is generally considered a favorable indicator for PD-1/PD-L1 inhibitor efficacy in NSCLC (12). However, the predictive ability of one single static indicator is limited. The patient also had TP53 mutations. TP53 mutations can be classified as “disruptive” (d) or “non-disruptive” (nd) according to their impact. TP53-d may have a predictive impact in localized-stage NSCLC, but further research is needed (13). Although TP53 alterations may correlate with genomic instability, PD-L1 expression or higher TMB in some NSCLC cohorts, TP53 mutation alone should not be considered an independent predictor of ICI benefit (14, 15). The tumor was MSS, which should likewise be interpreted as the absence of MSI-H. It has been proposed that immunotherapy failure in MSS is not merely a consequence of intrinsic immune indifference but instead reflects a hierarchical immune gating problem (16). But only a small percent of patients NSCLC have MSI-H, so MSS cannot be invoked as a standalone explanation for poor response (17). Based on the analysis results of the biomarkers in this case, it is clear that relying solely on a single static biomarker has its limitations. A comprehensive assessment of PD-L1, MSI, TP53, SMARCA4/2 alterations, KEAP1 and PTEN may be required to predict clinical benefit better (18). To address these issues, many researchers have already made attempts, but SD-NSCLC remains a therapeutically challenging subtype with no established standard targeted therapy.

The failure of first-line chemoimmunotherapy in this case also raises the question of whether ICI rechallenge can be effective after primary resistance. Current evidence suggests that ICI rechallenge tends to be more effective in patients who initially responded to ICI but later discontinued due to immune-related toxicity or stopped after completing a fixed treatment duration (19). In contrast, those with primary resistance have a lower likelihood of responding to ICI rechallenge. In our patient, therefore, the durable disease control observed after progression is unlikely explained by simple ICI rechallenge. Instead, it is may because LDRT provided a crucial immunomodulatory boost by remodeling the tumor microenvironment (TME). And given the patient’s MSS and TMB-low status, which is generally less predictive of ICI responsiveness, LDRT may have promoted immunogenic tumor-cell death and tumor-antigen release, thereby improved antigen presentation and immune recognition. The patient has achieved disease control for over 15 months, which is remarkable given that the highly aggressive nature of this molecular subtype. The reported median overall survival (OS) for the general SD-NSCLC population is only 12.2 months, and the prognosis for male patient is only 2.75 to 3.0 months (20). Our experience suggests that integrating systemic therapy with localized LDRT may benefit some specific patients.

In our patient, the rapid shrank of the large submandibular mass following LDRT suggests the possibility of a synergistic interaction between LDRT and ICI therapy. Based on previous literature and the clinical course we observed, we hypothesize that LDRT may have contributed to tumor control partly through immune modulation, although this was not directly demonstrated by experimental or paired tissue analyses in this case. Some studies suggest that, unlike high-dose radiotherapy, LDRT seems acts as an immunomodulator capable of changing the TME from an immunologically ‘cold’ or ‘immune-desert’ phenotype into a ‘hot’ state (21). Emerging evidence suggests that SD-NSCLC comprises both immune-desert and immune-enriched subtypes, which may partly explain the heterogeneous responses to immunotherapy observed in patients with SD-NSCLC. SMARCA4 deficiency may promote immune escape and primary resistance to PD-1 blockade by impairing CD8+ T-cell fitness through dysregulation of the IL-2/STAT5 signaling pathway. In addition, an immune-desert TME, together with co-occurring alterations such as KRAS, KEAP1, and STK11 mutations, may further compromise the efficacy of immunotherapy in this population. Previous studies suggest that LDRT can induces the polarization of tumor-associated macrophages (TAMs) toward a tumoricidal M1-like phenotype and promotes the abundant infiltration of effector cells, including CD8+ cytotoxic T lymphocytes (CTLs) and natural killer (NK) cells. Furthermore, while standard or higher doses of radiation often trigger the activation of latent TGFβ via reactive oxygen species—thereby promoting M2 macrophage differentiation and Treg cell recruitment—specific LDRT regimens have been shown to reduce the expression of immunosuppressive cytokines such as TGFβ1, which typically facilitates immune evasion (22). By avoiding TREX1 induction, LDRT may preserve cGAS–STING-mediated type I interferon signaling and thereby enhance the efficacy of immune checkpoint inhibitors (23). Hence, we speculate that LDRT may have helped reduce the immunosuppressive burden and maintain innate immune activation. However, this mechanistic interpretation remains hypothetical.

At present, the treatment of SD-NSCLC lacks universally accepted treatment guideline, largely owing to its marked molecular and clinical heterogeneity as well as the paucity of prospective evidence. The dependency created by SMARCA4 loss has prompted the development of synthetic-lethal strategies targeting the paralogous SWI/SNF ATPase SMARCA2. Several early-phase clinical trials have evaluated or are evaluating SMARCA2-directed agents in patients with advanced or metastatic SMARCA4-mutant solid tumors, including NSCLC. PRT3789 is a SMARCA2 degrader. It was used as a single-agent treatment and in combination with docetaxel in a phase 1 study (24). The oral SMARCA2 degrader PRT7732 has entered phase 1 clinical trials, but was terminated due to the decision of the sponsor (25). A phase 2 study of PRT3789 combined with pembrolizumab was also initiated to explore whether SMARCA2 degradation could be integrated with immune checkpoint blockade (26). But this trial was terminated early and no efficacy results have been posted. And LY4050784 is currently being evaluated in an ongoing phase 1 study of patients with advanced or metastatic SMARCA4/BRG1-altered solid tumors. In addition to monotherapy cohorts, this study has expanded to include combination regimens with pembrolizumab and platinum-based chemotherapy for patients with NSCLC. These trials indicate that molecular therapy for SMARCA4-altered tumors is popular field of investigation, but no definite results has been obtained yet. Against this background, the durable disease control achieved in our patient after the LDRT is remarkable, suggesting that modulation of the TME may offer a practical strategy when both conventional systemic therapy and new drugs approaches remain limited.

Likewise, the optimal application of LDRT remains undefined. Reported LDRT schedules have generally been explored within a low-fractionation range of 1–10 Gy and are most commonly combined with stereotactic body radiotherapy (SBRT) and/or ICIs (27). According to the current evidence, patients with SD-NSCLC beyond stage I are unlikely to derive durable survival benefit from surgery alone. Even more challenging, these tumors often exhibit poor responsiveness to conventional platinum-based chemotherapy and are prone to developing resistance to standard cytotoxic regimens. In this context, ICIs combined with LDRT have brought hope for the treatment of SD-NSCLC.

Patients with SD-NSCLC often require multimodal and sequential treatment strategies, and the timing and integration of chemotherapy, immunotherapy, and radiotherapy may be critical to clinical outcomes. And the SMARCA4-deficient has not only been reported in NSCLC, but also presents in various malignant tumors, including gastric cancer, ovarian cancer, uterine cancer, colorectal cancer, bladder cancer and breast cancer (28, 29). And in those research, SMARCA4 deficiency is always associated with rapid disease progression and poor clinical outcomes. In our case, the first-line immunotherapy failed to achieve disease control, which highlights the intrinsic heterogeneity and potential primary resistance of SD-NSCLC to immune checkpoint blockade. Against this background, LDRT may deserve particular attention, not as a tumor-eradicating modality, but as an immune-modulating intervention capable of reprogramming the TME. In previous studies, LDRT has been hypothesized to affect the TME, including T-cell infiltration and a potential shift from an immune-desert phenotype toward a more inflamed state. But these mechanisms were not directly demonstrated in our patient. However, based on our clinical experience with this case, we believe that incorporating low-dose radiotherapy into the sequential treatment plan may be worthy of further investigation.

Safety data was also reviewed because the patient received repeated LDRT together with systemic therapy. Intervention adherence and tolerability was assessed by reviewing treatment records and consulting the patient. No grade 4 or grade 5 adverse events were recorded. The treatment did no paused because of adverse events, and the toxicities were clinically manageable (**Table 2**).

**Table 2:** Treatment-related adverse events.

Treatment start date	Treatment regimen	Anemia	Platelet count decreased	Leukopenia	Neutropenia	ALT or AST increased	Pneumonia	Nausea	Vomiting	Diarrhea	Constipation	Oral pain	Mucositis oral	Creatinine increased	Rash	Hemorrhage
07/02/2025	Serplulimab + EP	1	—	1	1	1	—	1	—	—	—	—	—	1	—	—
04/03/2025	LDRT	1	—	—	—	—	—	—	—	—	—	—	—	—	—	—
12/03/2025	Serplulimab + EP	1	—	2	2	—	—	1	—	—	—	1	1	—	—	—
03/04/2025	Serplulimab + EP	1	—	2	2	—	—	1	1	—	—	—	—	1	—	—
14/04/2025	LDRT	1	1	1	1	—	—	—	—	—	—	—	—	—	—	—
24/04/2025	Serplulimab + EP	1	—	1	1	—	—	1	—	—	—	—	—	1	—	—
27/05/2025	LDRT, Serplulimab + EP	1	—	2	3	—	—	1	1	—	—	—	—	1	—	—
05/07/2025	Serplulimab + EP	1	—	—	—	—	—	—	—	—	—	—	—	1	—	—
09/07/2025	LDRT	1	1	2	2	—	—	1	—	—	—	1	1	1	—	—
28/08/2025	LDRT	—	—	—	—	—	—	1	1	—	—	—	—	0	—	—
10/09/2025	Serplulimab	1	—	2	2	—	—	1	—	—	—	—	—	1	—	—
17/10/2025	LDRT	—	—	—	—	—	—	—	—	—	—	—	—	1	—	—
05/12/2025	LDRT	—	—	—	—	—	—	—	—	—	—	—	—	1	—	—
22/12/2025	Serplulimab	—	—	2	2	—	—	1	1	—	—	—	1	1	—	—
16/01/2026	LDRT	—	—	1	1	—	—	—	—	—	—	—	1	1	—	—
23/01/2026	Serplulimab	—	—	—	1	—	—	—	—	—	—	—	—	1	—	—

EP, etoposide/cisplatin.

This study has several limitations. First, as a single case report with multiple combined interventions, it is inherently difficult to disentangle the contribution of each modality to the overall therapeutic effect. Second, further preclinical and clinical investigations are therefore warranted to elucidate the specific mechanisms underlying the efficacy of LDRT in SD-NSCLC, and to identify reliable biomarkers that can define the patient subgroups most likely to benefit from this approach. In addition, the follow-up duration is relatively short; extended observation over a longer period is necessary to more accurately assess the durability of clinical responses and the long-term safety profile of this treatment strategy.

## Conclusion

SD-NSCLC is an aggressive molecular subtype with limited therapeutic options and frequent resistance to standard systemic therapy. In this case, LDRT may have contributed to disease control as part of multimodal treatment, but its independent effect remains uncertain. Further studies are needed to clarify the optimal role of LDRT in the multimodal management of SD-NSCLC.

## Patient perspective

According to the patient and his family, the rapid disease progression caused substantial anxiety and their concern about prognosis. The visible gingival and cervical lesions also affected his daily comfort and contributed to emotional distress. After salvage treatment, the reduction of the visible lesions provided him reassurance and improved his confidence in continuing therapy. At the latest follow-up, the patient remained clinically stable and reported that his overall condition was acceptable. He told us, the control of tumor allowed him to regain a sense of normalcy in daily life, feeling less like a patient defined by his disease. Meanwhile, he emphasized that maintaining disease control, treatment tolerability, and quality of life remained his concerns.

The patient continues to attend regular follow-up visits at our hospital, actively cooperates with scheduled examinations.

## Data Availability

The original contributions presented in the study are included in the article/**Supplementary Material**. Further inquiries can be directed to the corresponding author.

## Supplementary material

Supplementary Figure 1 Representative pathological and immunohistochemical images. **(A)** primary tumor HE, **(B)** gingival metastasis 10×10 HE, **(C)** CK7 gingival metastasis 10×10 IHC.

Supplementary Figure 2 **(A)** CT image of metastatic lesion obtained on November 28, 2025. **(B)** CT image of metastatic lesion obtained on March 2, 2026. **(C)** CT image of metastatic lesion obtained on June 2, 2026.
